# Effect of gadolinium concentration on temperature change under magnetic field

**DOI:** 10.1371/journal.pone.0214910

**Published:** 2019-04-04

**Authors:** Kemal Arda, Sinan Akay, Cevat Erisken

**Affiliations:** 1 Health Sciences University, Gulhane Medicine Faculty, Research and Education Hospital, Department of Radiology, Ankara, Turkey; 2 Nazarbayev University, Chemical and Materials Engineering, Astana, Kazakhstan; Henry Ford Health System, UNITED STATES

## Abstract

Gadolinium based contrast agents (GBCAs) were found to play a role in nephrogenic systemic fibrosis in patients with and without renal impairment. Therefore, preserving the structural stability of GBCAs to reduce their propensity to liberate Gd^3+^ is of utmost importance. This study evaluates the effect of gadolinium concentration of GBCAs on solution temperature under magnetic fields. It is hypothesized that presence of gadolinium will lead to temperature changes of its solutions under magnetic field, and this change will depend on concentration. In this study, GBCAs were diluted to concentrations of 0.6, 1.2, 1.8, 2.4 mMol/L. A 10mL preparation in pure water, simulated body fluid (SBF), and plasma was scanned at 3T following a soft tissue neck protocol, and their temperatures were measured. Findings revealed that concentration of GBCA had significant effect on temperature change in all dilution media. Type of commercially available GBCA had an effect only in SFB and plasma. Evaluation of correlation between conditional stability constant (K_cond_) and temperature difference (ΔT) revealed that in water and SBF there exists a positive correlation between K_cond_ and temperature variation. Collectively, GBCAs can cause local temperature variations when administered into patients, and can affect dissociation of gadolinium from its chelates, which should be investigated in a further study.

## Introduction

Gadolinium chelates are preferred in magnetic resonance imaging as contrast agents due to their capacity to improve visibility. Such chelates, also known as Gadolinium-based contrast agents (GBCAs), were previously known to be safely and dose-dependently tolerated. For example, single intravenous administrations of Gadobutrol up to 0.5 mmol per kilogram of body weight were reported to be well tolerated.[1] Free ionic gadolinium (Gd^3+^) is, on the other hand, extremely toxic. In addition, since ionic radii of Gd^3+^ and Ca^2+^ are close to one another, Gd^3+^ may act as an inorganic blocker of calcium channels at nano- to micro-molar concentration levels.[2] Chelation of gadolinium by ligands was shown to decrease its acute toxicity to acceptable ranges.[3] Therefore, preserving the structural stability, more specifically kinetic and thermodynamic stability, of these chelates to reduce their propensity to liberate Gd^3+^ is of utmost importance. Recently, GBCAs were found to play a role in the progression of nephrogenic systemic fibrosis (NSF) in patients with severe renal impairment, and this role was more recently found to extend to accumulation of Gd^3+^ in tissues of patients with no renal impairment.

Chemical structure of ligands of GBCAs available in the market is either open-chain or macrocyclic, and the chelates formed by association of such ligands with gadolinium are either ionic or nonionic.[4] Thermodynamic stability of a gadolinium complex is determined by the equilibrium constant, and defines the equilibrium concentrations of all species in a solution. Equilibrium state is given by the following equation[5]:

$${Gd(H_{2}O)}_{8}^{3+}+L↔GdL(H_{2}O)+7H_{2}O$$ (1)

The relevant thermodynamic stability constant is then calculated by:

$$K_{st}=\frac{\left[GdL\right]}{\left[{Gd}^{3+}][L\right]}$$ (2)

where terms in the brackets represent molar concentrations of the complex [GdL], ligand [L] and gadolinium [Gd^3+^] under equilibrium conditions.

A more effective constant for stability is the conditional stability constant. This better represents the stability of a complex because thermodynamic stability constant assumes that the ligand is completely deprotonated, which in fact is not the case under physiologic conditions (pH ~7.4). Therefore, following equilibrium reaction, and the relevant equation for stability constant (*K*_*eff*_) more correctly predict the stability of chelates under physiologic conditions.

$${Gd(H_{2}O)}_{8}^{3+}+H_{n}L↔GdL(H_{2}O)+nH^{+}+7H_{2}O$$ (3)

$$K_{eff}=\frac{[GdL]{\left[H^{+}\right]}^{n}}{\left[Gd][H_{n}L\right]}$$ (4)

Where HnL is the protonated form of free ligands.

Properties of some of the clinically used GBCAs are provided in Table 1. From this table, it can be clearly inferred that stability of GBCAs varies depending on their structure (macrocycle or linear/open-chain) and charge. Stability of macrocyclic chelates is also very much related to the presence of macrocycle bridges between two metal centers.[6]

**Table 1:** Properties of some commercial gadolinium based chelates†.

Generic Name	Gadoteric acid meglumine	Gadobutrol	Gadoteridol	Gadoxetic acid disodium salt	Gadoversetamide	Gadodiamide
Acronym	Gd-DOTA	Gd-BT-DO3A	Gd-HP-DO3A	Gd-EOB-DTPA	Gd-DTPA-BMEA	Gd-DTPA-BMA
**Chemical Structure**	Macrocyclic	Macrocyclic	Macrocyclic	Open-chain	Open-chain	Open-chain
**Charge**	Ionic	Nonionic	Nonionic	Di-ionic	Nonionic	Nonionic
**Kinetic stability** *	High	High	High	Medium	Low	Low
**Thermodynamic stability constant (logK_therm_)**	25.6	21.8	23.8	23.5	16.6	16.9
**Conditional stability constant (log K**_**cond**_**)**	19.3	14.7	17.1	18.7	15.0	14.9
**Dissociation rates (t**_**1/2**_**) in 0.1M HCl (25°C, pH = 1)**	338 h/9.2 h**	43h/24h***	3.9h	<5sec/9.6 min**	<5sec	<5sec/< 34 s**
**Concentration of commercial product (M)**	0.5	1.0	0.5	0.25	0.5	0.5
**Formulation (mMol/L)**	No free ligand added	1.0	0.5	1.5	50	25

† Adapted from Ideeet al 2009

* Low: long-time index<0.3; Medium: 0.3<long-time index<0.95; High: long-time index>0.95

** Sherry et al 2009

*** Hanedar et al 2015

In one extensive study, low-K_therm_gadodiamide was compared to high-K_therm_ gadoteridol in cultured fibroblasts and rats with uninephrectomies.[7] Study found negligible differences the two contrast between agents in terms of toxicity or proliferation. In vivo, greater skin fibrosis and dermal cellularity were observed with gadodiamideas compared to gadoteridol treatment. In addition, fibrosis due to contrast agent appeared to be limited to skin and kidney. Overall, the study concluded that low-K_therm_ chelates have a greater propensity to provoke nephrogenic systemic fibrosis.

Although there are abundant studies for the determination of thermodynamic stability of the GBCAs, the effect of temperature on stability or dissociation kinetics of GBCAs has not been investigated. Early reports on the temperature dependency of dissociation constants of cadmium,[8] zinc, and copper complexes[9] reveal a positive correlation between temperature and dissociation constant. Therefore, dissociation of Gd^3+^ from its chelates may very well be dependent on the local as well as entire body temperature in addition to structure and charge of the chelates, and pH of medium. However, to the best of our knowledge, literature is deficient in terms of this potential contribution from temperature changes to the toxicity of GBCAs.

The magnetocaloric effect (MCE) is an event leading to reversible variations in temperature of the material when exposed to a changing magnetic field. It is a result of the effect of external magnetic field on isothermal entropy and adiabatic temperature[10], and is defined by the thermodynamic Maxwell relation[11]:

$${\left(\frac{\partial S}{\partial B}\right)}_{T}={\left(\frac{\partial M}{\partial T}\right)}_{B}$$ (5)

where S is entropy, B is the magnetic field, M is magnetization and T is temperature.

Paramagnetic properties of gadolinium are due to the presence of unpaired electrons, and arise from the realignment of the electron paths governed by the applied external magnetic field. As the atoms become more aligned, resulting with reduced entropy of the system, the material's temperature rises.[12] Upon removal of the magnetic field, the electron paths become unaligned, which eventually leads to cooling of the material.[11] During this heating and cooling period, heat is exchanged between the gadolinium based contrast agent and its surroundings.

Magnetic field leads to changes in paramagnetic material’s temperature. It could, then, be claimed that accumulation of gadolinium in tissues could also affect behavior of host cells in the neighborhood. In this regard, one recent study demonstrated that the pulsed heat shocks (at 45°C and 60°C) enhance procollagen type I and procollagen in type III expression human dermal fibroblasts.[13]

Based on the published data about temperature dependency of dissociation constants, dissociation of Gd^3+^ from its chelates, and the resultant accumulation in tissues may be related to local temperature changes. Therefore, this study aims at evaluating the effect of gadolinium concentration on solution temperature upon entering and leaving magnetic fields so as to simulate the case of a patient, injected with a GBCA, entering and leaving magnetic fields. Although it is out of scope of this study, such dependency should motivate researchers to study temperature dependency of dissociation kinetics as well as thermal effects on the thermodynamic stability of GBCAs.

It hypothesized that presence of gadolinium will lead to temperature changes of its solutions when exposed to magnetic field, and that the change will depend on the concentration of gadolinium in the solution.

## Methods

### Contrast agent dilutions

Each of five GBCAs, namely, Gadoteric acid meglumine (Dotarem, Guerbet, Roissy CDG, France), Gadobutrol (Gadovist, Bayer HealthCare, Berlin, Germany), Gadodiamide (Omniscan, GE HealthCare, AS Oslo, Norway), Gadoversetamide (Optimark, Covidien, Mallinckrodt Inc., North Carolina, USA), and Gadoxetic acid disodium salt (Primovist, Bayer HealthCare, Berlin, Germany) were diluted in pure water, simulated body fluid (SBF, 0.9% NaCl (Cetinkaya Pharmaceutical Industry and Trade Inc., Bolu, Turkey) and plasma (obtained from two patients with consent, K.N.A and S.A) to yield final concentrations of 0.6, 1.2, 1.8, 2.4 mMol/L. A total of 10mL preparation from each GBCA was transferred to cylindrical tubes, implemented to MRI device (Achieva, 3.0 T X-Series, Phillips Medical Systems, Best, The Netherlands), and their temperatures were measured using an MRI compatible thermocouple (Neoptix, Isha Surgical, Kamataka, India). These GBCAs are marketed at concentrations provided in Table 1, in preparations with predetermined concentrations to inject intravenously.

### MRI parameters

Samples were implemented to a 3T Philips Achieva MRI Scanner and exposed to 3T magnetic flux density. A total of eight sequences was employed to scan the solution: Survey, Ref NV-16, STIR-TSE-COR, T2W-TSE-SAG, T2W-SPRR-TRR, T1W-TSE-TRR, T1-TSE-SPR-TRR, and SDWI-TRR. The solution was exposed to magnetic flux at all times, starting from implementation of solution to MRI to the end of scan. The GBCAs contained in the tubes were treated as patients undergoing MRI scans with a soft tissue neck protocol. Details of scan parameters at each sequence are provided in Table 2.

**Table 2:** Sequences and scan parameters employed in MRI scans.

Sequence	Parameter
Survey:	TR/TE: 49/1.49 ms, ACQ matrix MxP: 256x179
Ref NV-16:	TR/TE: 4/0.59 ms, ACQ matrix MxP: 56x40
STIR-TSE-COR:	TR/TI: 5129/180 ms, TE: 15 ms, ACQ matrix MxP: 196x101, FOV RL-AP: 210–167 mm, Slice Thickness: 4 mm, Recon matrix: 256, SENSE: Yes, Slices: 30
T2W-TSE-SAG:	TR: 3610 ms, TE: 80 ms, ACQ matrix MxP: 240x168, FOV RL-AP: 210–196 mm, Slice Thickness: 4 mm, Recon matrix: 432, SENSE: Yes, Slices: 30
T2W-SPRR-TRR:	TR: 3857 ms, TE: 59 ms, ACQ matrix MxP: 276x1689, FOV RL-AP: 250–199 mm, Slice Thickness: 4 mm, Recon matrix: 512, SENSE: Yes, Slices: 30
T1W-TSE-TRR:	TR: 1077 ms, TE: 9.2 ms, FOV RL-AP: 250–199 mm, Slice Thickness: 4 mm, Recon matrix: 512, SENSE: Yes, Slices: 30
T1W-TSE-SPR-TRR:	TR: 1149 ms, TE: 9.2 ms, ACQ matrix MxP: 256x139, FOV RL-AP: 250–199 mm, Slice Thickness: 4 mm, Recon matrix: 512, SENSE: Yes, Slices: 30
SDWI-TRR:	TR: 1149 ms, TE: 9.2 ms, ACQ matrix MxP: 256x139, FOV RL-AP: 230 mm, Slice Thickness: 4 mm, Recon matrix: 256, SENSE: Yes, Slices: 30

### Temperature measurements

A probe was inserted in the tube entering magnetic field, and is connected to a measuring device located outside MRI room at a distance of approximately 10m. Temperature readings were recorded at the end of each sequence given in Table 2.

### Statistical analysis

Data were analyzed for the effect of concentration of GBCA and dilution medium (pure water, simulated body fluid, and plasma) on temperature variation of the solution. A two-way analysis of variance (ANOVA) was used to determine the effects of concentration of GBCA and dilution medium on temperature change. Bonferroni post hoc test was used for all pairwise comparisons, and significance was attained at p < 0.05. Statistical analyses were performed with Prism (Version 5.0, GraphPad Software, La Jolla, CA).

## Results

The effect of GBCA concentration on temperature variation was analyzed in two different ways. One is temperature variation between each sequence (ΔT_sequence_), and the other is temperature variation recorded between the time the GBCA is implemented in MRI and the time it was removed (ΔT_overall_). In the first analysis, temperature variation between each sequence was taken as a single data point, which yielded a total of seven data points (n = 7), while in the second one there is only one data point, which is the difference between entrance and exit temperature. Statistical analysis was, therefore, performed only for the first method.

### Effect of GBCA concentration and type on temperature change within sequences in different dilution media

**Fig 1:**
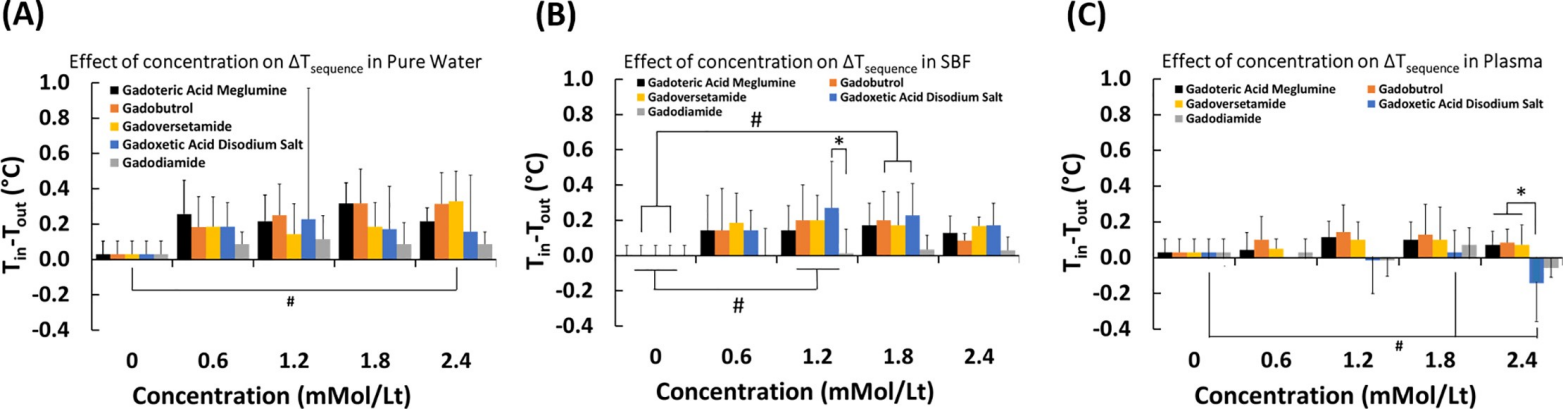
Effect of GBCA concentration and type on temperature change within sequences in different dilution media. (A) pure water, (B) Simulated body fluid-SFB, (C) plasma. Concentrations: 0.6, 1.2, 1.8, 2.4 mMol/L.

Effect of GBCA concentration on temperature change within sequences (ΔT_sequence_) was evaluated for solutions prepared in pure water, SBF, and plasma (Fig 1). For the solutions prepared in pure water, statistical analysis yielded that there is no effect of GBCA type on temperature, while concentration is found to have a significant effect on temperature (Fig 1A). In addition, no interaction between these two is present. The only significant effect of concentration is that under 3T magnetic field, Gadoversetamide with 2.4mMol/L concentration in pure water led to higher temperature drop as compared to pure water, i.e., 0 mMol/L (p<0.05, indicated by the # sign).

For the solutions prepared in SBF, statistical analysis yielded that there is an effect of both GBCA type and concentration on temperature (Fig 1B). No significant interaction between these two is present. In terms of the effect of GBCA type, post-hoc test revealed that at 1.2mMol/L concentration level, Gadoxetic acid disodium salt led to significantly higher (p<0.01) temperature drop relative to Gadodiamide. Regarding the effect of concentration, Gadobutrol, Gadoversetamide, and Gadoxetic acid disodium salt solutions led to significantly higher (p<0.01) temperature drops at 1.2mMol/L concentration level as compared to 0mMol/L level. In addition, both Gadobutrol and Gadoxetic acid disodium salt had higher temperature drops (p<0.05) at 1.2mMol/L concentration level as compared to 0mMol/L level.

For the solutions prepared in plasma, statistical analysis yielded that there is an effect of both GBCA type and concentration on temperature (Fig 1C). No significant interaction between these two is present. In terms of the effect of GBCA type, post-hoc test revealed that at 2.4mMol/L concentration level, Gadoteric acid meglumine, Gadobutrol and Gadoversetamide led to significantly higher (p<0.01) temperature drop relative to Gadoxetic acid disodium salt, of which temperature actually increased. Regarding the effect of concentration, Gadoxetic acid disodium salt solutions led to significantly higher (p<0.05) temperature drops at 2.4mMol/L concentration level as compared to 0mMol/L and 1.8mMol/L levels.

### Correlation between conditional stability constant (K_cond_) and temperature variation within a sequence (ΔT_sequence_) in different media

**Fig 2:**
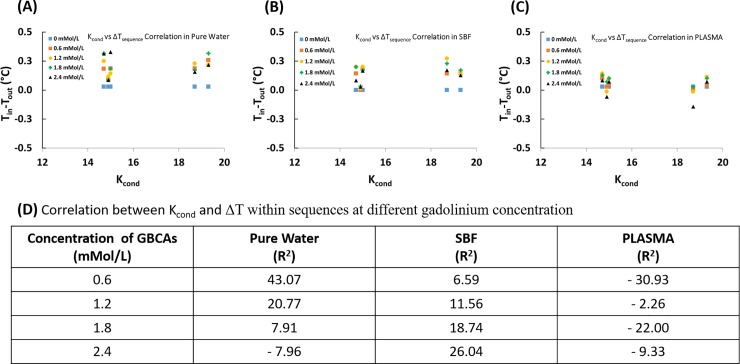
Scatter plots showing correlation between conditional stability constant (K_cond_) and temperature difference (ΔT_sequence_) within a sequence in different media. (A) pure water, (B) Simulated body fluid-SFB, (C) plasma, and (D) relevant correlation coefficients.

Conditional stability constants for GBCAs used for correlation determination were taken from previous studies (Table 1). Specifically, the conditional stability constants (log K_cond_) for Gadoteric acid meglumine, Gadobutrol, Gadoxetic acid disodium salt, Gadoversetamide, and Gadodiamide are 19.3, 14.7, 18.7, 15.0, and 14.9, respectively. Since this property defines the propensity of Gd^3+^ to dissociate from its chelate, any correlation between stability and temperature change would provide valuable information about the effect of presence of Gd^3+^ on temperature change. Fig 2 provides scatter plots for temperature variation as a function of K_cond_, and the resultant R^2^ values (R^2^ = 100 represents perfect correlation).

Based on correlation coefficients (Fig 2D), temperature variation in GBCA solution in pure water when exposed to 3T magnetic field could be related to conditional stability constant to the extent of 43.07% when prepared at a concentration of 0.6mMol/L. In other concentration levels, correlation is 20.77% or lower. In SBF, temperature variation in GBCA solution could be related to conditional stability constant to the extent of 26.04% when prepared at a concentration of 2.4mMol/L. In other concentration levels in SBF, correlation is 18.74% or lower. In plasma, however, temperature variation in GBCA solution could be related to conditional stability constant to the extent of 30.93% when prepared at a concentration of 0.6mMol/L. In other concentration levels in plasma, correlation is 22% or lower.

### Effect of GBCA concentration and type on temperature variation within the entire process in different dilution media

**Fig 3:**
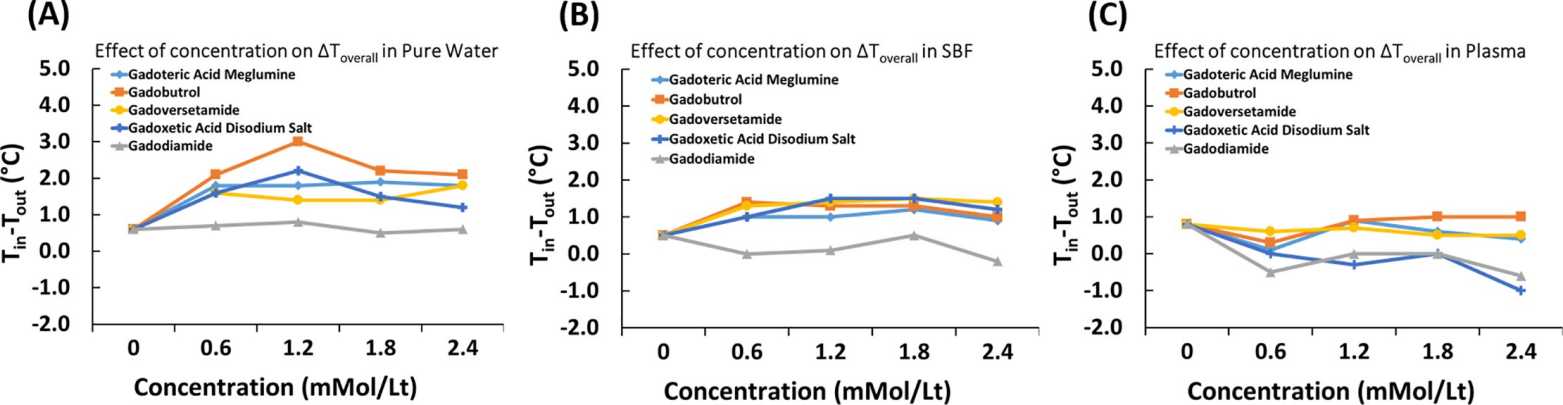
Effect of GBCA concentration and type on temperature change within the entire process in different dilution media. (A) pure water, (B) Simulated body fluid-SFB, (C) plasma. Concentrations: 0.6, 1.2, 1.8, 2.4 mMol/L.

Effect of GBCA concentration on temperature change through the entire process (ΔT_overall_) was evaluated for solutions prepared in pure water, SBF, and plasma (Fig 3). No statistical analysis was performed for the data because there is only one data point, which is the difference between entrance and exit temperature. In pure water, the highest and lowest temperature drop was observed for Gadobutrol and Gadodiamide, respectively, at all concentration levels. Gadoteric acid meglumine, Gadoversetamide, and Gadoxetic acid disodium salt showed fluctuations in the concentration range between 0mMol/L to 2.4 mMol/L. In SBF, the lowest temperature drop was observed for Gadodiamide at all concentration levels. Other GBCAs exhibited fluctuations in the concentration range between 0 mMol/L to 2.4 mMol/L. In plasma, the lowest temperature drop was observed for the group of Gadoteric acid meglumine and Gadodiamide at all concentration levels. In fact, temperature of solutions for Gadoteric acid meglumine and Gadodiamide increased under magnetic field. The highest temperature drop was generally seen for Gadobutrol, Gadoversetamide, and Gadoxetic acid disodium salt in the concentration range between 0 mMol/L to 2.4 mMol/L.

### Correlation between conditional stability constant (K_cond_) and temperature difference (ΔT_overall_) in entire process in different media

**Fig 4:**
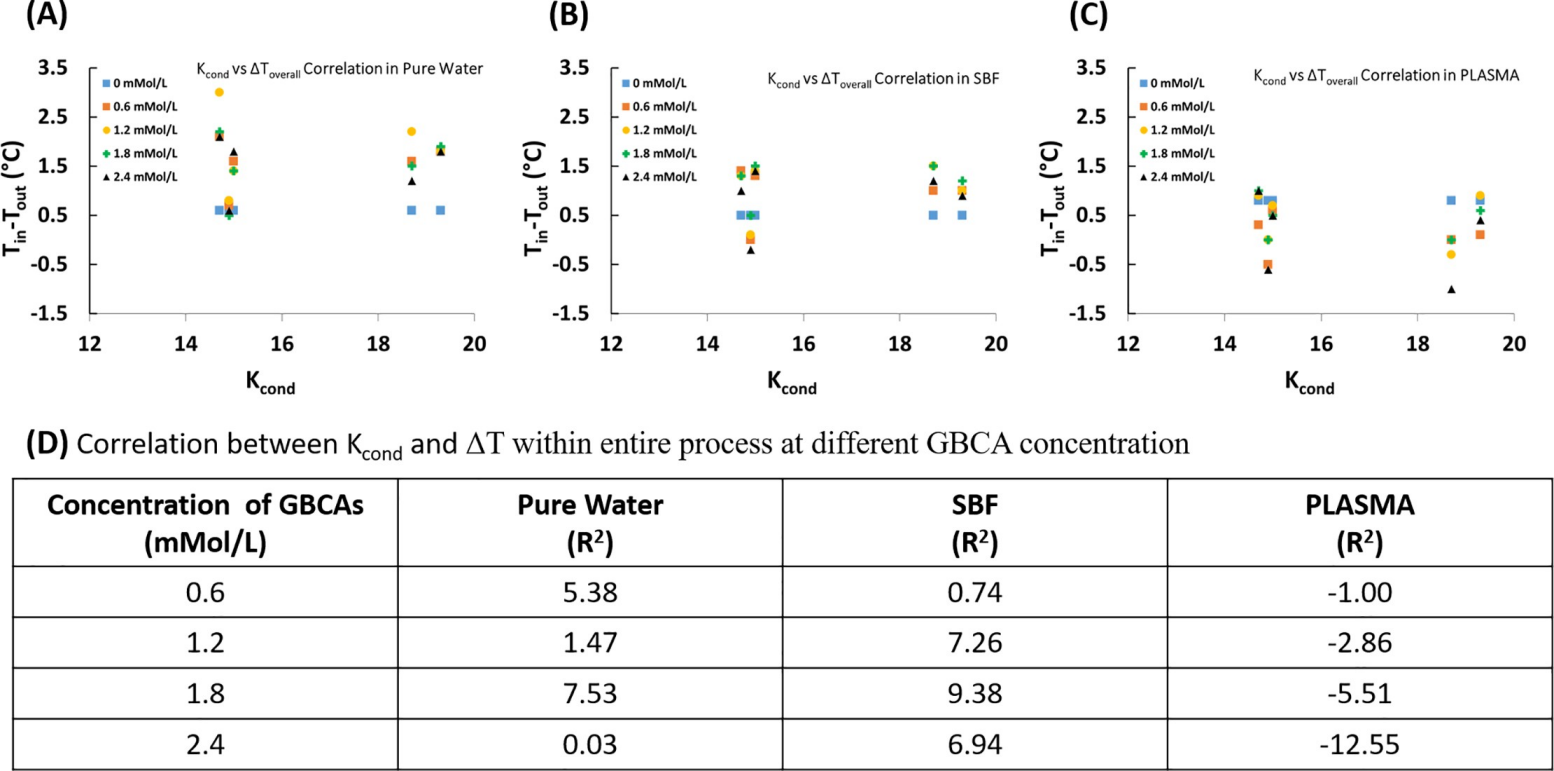
Scatter plots showing correlation between conditional stability constant (K_cond_) and temperature difference (ΔT_overall_ in entire process in different media. (A) pure water, (B) Simulated body fluid-SFB, (C) plasma), and (D) relevant correlation coefficients.

Fig 4 provides scatter plots for temperature variation as a function of K_cond_, and the resultant R^2^ values (R^2^ = 100 represents perfect correlation).

Based on correlation coefficients (Fig 4D), temperature variation in GBCA solution in pure water when exposed to 3T magnetic field could be related to conditional stability constant to the extent of 7.53% when prepared at a concentration of 1.8mMol/L. In other concentration levels, correlation is approximately 5% or lower. In SBF, temperature variation in GBCA solution could be related to conditional stability constant to the extent of 9.38% when prepared at a concentration of 1.8mMol/L. In other concentration levels in SBF, correlation is approximately 7% or lower. In plasma, temperature variation in GBCA solution could be related to conditional stability constant to the extent of 12.55% when prepared at a concentration of 2.4mMol/L. In other concentration levels in SBF, correlation is 5.5% or lower.

### Relation between temperature variations and paramagnetic properties (concentration) of gadolinium

**Fig 5:**
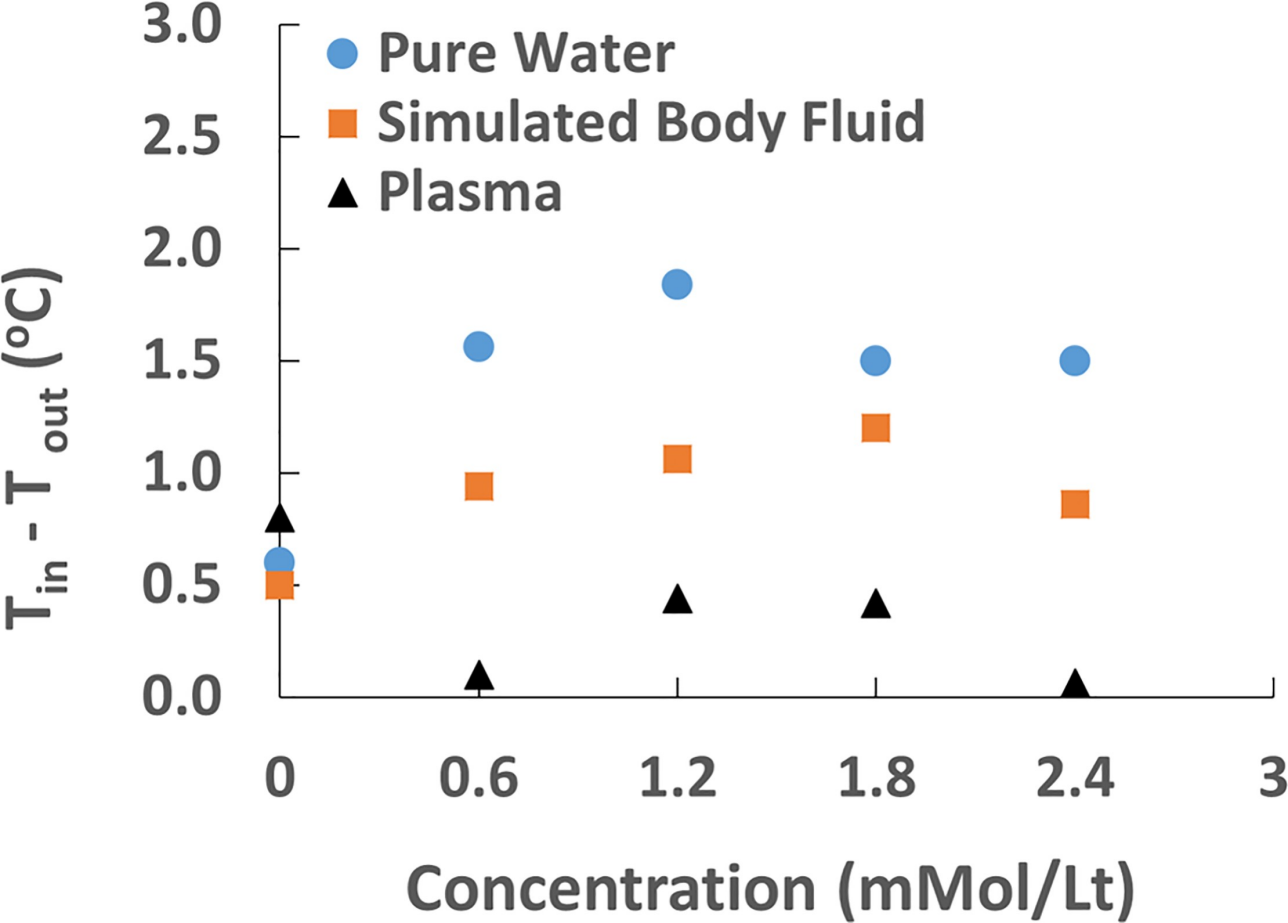
Scatter plots showing correlation between temperature difference (ΔT_overall_) and concentration in different media.

Gadolinium exhibits paramagnetic properties due to the presence of unpaired electrons, and these electrons realign under external magnetic field leading to an increase in the material's temperature. The effect of concentration on temperature change was also studied and the results are illustrated in Fig 5. Apparently, no correlation existed between concentration and temperature change in different media.

## Discussion

Gadolinium-based chelates are still the most preferred contrast agents in MRI despite their proven (dose-dependent) accumulation in different tissues of patients with no renal impairment and role in the progression of nephrogenic systemic fibrosis (NSF) in patients with severe renal impairment. Their effect is now more closely monitored, and intense investigations are in place to understand its safety [14,15,16] and the mechanism of dissociation of Gd^3+^ from its chelates including the effect of structure and charge of the chelates, and pH of the medium. [5,14,15,17]

In addition to structure and charge of the chelates, and pH of the medium, local temperature can also have significant effects on the dissociation of Gd^3+^ from its chelates, and the resultant accumulation in tissues. This was experimentally shown for other complexes including cadmium,[8] zinc, and copper[9], for which a positive correlation between temperature and dissociation constant was shown. However, the pool of literature on this topic does not provide any clear evidence in terms of this potential contribution from temperature changes to the toxicity of GBCAs.

Here, we evaluated, in vitro, the effect of gadolinium concentration on the variation of gadolinium containing solution temperature upon entering and leaving magnetic fields. This is to simulate the effect of magnetic field on the local temperature change in the presence of GBCAs. It is expected that presence of gadolinium will lead to temperature changes of its solutions when exposed to magnetic field, and that the change will depend on the concentration of gadolinium in the solution.

Effect of GBCA concentration on temperature change was evaluated for solutions prepared in pure water, SBF, and plasma. Generally, concentration of GBCA had significant effect on temperature change in all dilution media. Type of commercially available GBCA had an effect only in SFB and plasma. Specifically, at 1.2mMol/L concentration in SBF, Gadoxetic acid disodium salt led to higher temperature drop as compared to Gadodiamide. In plasma, on the other hand, at 2.4mMol/L concentration Gadoteric acid meglumine, Gadobutrol, and Gadoversetamide led to higher temperature drop as compared to Gadoxetic acid disodium salt. Based on the entrance and exit temperature differences, Gadodiamide in water and SBF, and Gadodiamide and Gadoxetic acid disodium salt in plasma had the lowest temperature variation. These findings imply that in water and SBF at concentrations between 0.6mMol/L and 2.4mMol/L, all GBCAs (with an exception of Gadodiamide at specific concentrations) will lead to local temperature drops (cooling) when exposed to magnetic field with a magnitude of 3T. In plasma, on the other hand, Gadoxetic acid disodium salt and Gadodiamide at concentrations between 0.6mMol/L and 2.4mMol/L will lead to local temperature increase (warming) when exposed to magnetic field with a magnitude of 3T. Based on stability constants of these two (Table 1), such a finding seems interesting because Gadoxetic acid disodium salt (K_cond_ = 18.7) and Gadodiamide (K_cond_ = 14.9) stand at the opposite edges of the scale of stability constants of GBCAs studied here. In any case, the effect of temperature variation on the behavior of tissue specific cell lineages should be studied because dissociation and accumulation of gadolinium in tissues could affect behavior of host cells as demonstrated in the case of enhanced procollagen type I and procollagen type III expression in human dermal fibroblasts due to temperature variations.[13]

Evaluation of correlation between conditional stability constant (K_cond_) and temperature difference (ΔT) revealed that in water and SBF there exists a positive correlation between K_cond_ and temperature variation. This means that as K_cond_ increases temperature drop also increases, which brings about lower exit temperature (cooling) of the solution. In plasma, however, there exists a negative correlation between K_cond_ and temperature variation. And, this implies higher temperature of the solutions at the exit (warming). With reference to commercially available GBCAs, Gadodiamide and Gadoversetamide have lower stability constants, while Gadoteric acid meglumine and Gadobutrol possess higher stability constants (Table 1). Therefore, it is possible to claim,in the concentration range studied here, that when administered in or come into contact with plasma, Gadoteric acid meglumine and Gadobutrol can lead to increased local temperatures. However, how this increase in temperature will affect the dissociation of Gd^3+^ from its chelate still requires further investigation. It should also be noted that some GBCAs such as Gadoversetamide and Gadodiamide are formulated at concentrations of 50mMol/L and 25 mMol/L, respectively, and that they can behave differently as their concentrations fall out of the range studied here.

Gadolinium exhibits paramagnetic properties due to the presence of unpaired electrons, and these electrons realign under external magnetic field leading to an increase in the material's temperature.[12] Upon removal of the magnetic field, the electron paths become unaligned, which eventually leads to cooling of the material.[11] In this study, it was not possible to relate the temperature variations to paramagnetic properties (concentration) of gadolinium. This may be because of the extent of dilution. In a most relevant study, Hijnen et al[18] studied the effect of a paramagnetic MRI contrast agent (Gd-DTPA, Magnevist) in the tissue on proton resonance frequency shift (PRFS) temperature mapping. In a rat model, they found that intravenous Gd-DTPA injection (0.6 mmol/kg, 0.5 M, injection speed of 1 mL/min) resulted in a change of the local magnetic field, which translated into an apparent temperature change of −2.0°C ± 0.1°C(mean ± SD) in the middle of the hind leg muscle within approximately 2 min after injection. More recently Deka et al [19] determined the effect of gadolinium concentration in the formation biphasic α-Fe2O3-GdFeO3 for hyperthermia applications. The heating ability of the materials was tested under an alternating magnetic field and samples exhibited a rise in temperature in the range of hyperthermia temperature with a maximum temperature of 55.71°C in 6 min.

## Conclusion

This study aimed at evaluating the effect of gadolinium concentration and type of GBCA on solution temperature upon entering and leaving magnetic fields, simulating conditions a patient is undergoing during an MRI scanning. The effect of GBCA concentration on temperature change was evaluated for solutions prepared in pure water, SBF, and plasma. Generally, concentration of GBCA had significant effect on temperature change in all dilution media. Type of commercially available GBCA had an effect only in SFB and plasma. Evaluation of correlation between conditional stability constant (K_cond_) and temperature difference (ΔT) revealed that in water and SBF there exists a positive correlation between K_cond_ and temperature variation. Based on these findings, it can be concluded that the type of GBCAs can cause local temperature variations when administered into patients, and can affect dissociation of gadolinium from its chelates leading to potential toxic effect, which should be investigated in a further study. It is anticipated that results of this study will motivate researchers to study temperature dependency of dissociation kinetics as well as thermal effects on the thermodynamic/conditional stability of GBCAs.
